# Nudging Health Care Providers’ Adoption of Clinical Decision Support: Protocol for the User-Centered Development of a Behavioral Economics–Inspired Electronic Health Record Tool

**DOI:** 10.2196/42653

**Published:** 2023-01-18

**Authors:** Safiya Richardson, Katherine Dauber-Decker, Jeffrey Solomon, Sundas Khan, Douglas Barnaby, John Chelico, Michael Qiu, Yan Liu, Devin Mann, Renee Pekmezaris, Thomas McGinn, Michael Diefenbach

**Affiliations:** 1 New York University Grossman School of Medicine New York, NY United States; 2 Feinstein Institutes for Medical Research Northwell Health Manhasset, NY United States; 3 Baylor College of Medicine Houston, TX United States; 4 CommonSpirit Health Chicago, IL United States

**Keywords:** health informatics, clinical decision support, electronic health record, implementation science, behavioral economics, user-centered design, pulmonary embolism

## Abstract

**Background:**

The improvements in care resulting from clinical decision support (CDS) have been significantly limited by consistently low health care provider adoption. Health care provider attitudes toward CDS, specifically psychological and behavioral barriers, are not typically addressed during any stage of CDS development, although they represent an important barrier to adoption. Emerging evidence has shown the surprising power of using insights from the field of behavioral economics to address psychological and behavioral barriers. *Nudges* are formal applications of behavioral economics, defined as positive reinforcement and indirect suggestions that have a nonforced effect on decision-making.

**Objective:**

Our goal is to employ a user-centered design process to develop a CDS tool—the pulmonary embolism (PE) risk calculator—for PE risk stratification in the emergency department that incorporates a behavior theory–informed nudge to address identified behavioral barriers to use.

**Methods:**

All study activities took place at a large academic health system in the New York City metropolitan area. Our study used a user-centered and behavior theory–based approach to achieve the following two aims: (1) use mixed methods to identify health care provider barriers to the use of an active CDS tool for PE risk stratification and (2) develop a new CDS tool—the PE risk calculator—that addresses behavioral barriers to health care providers’ adoption of CDS by incorporating nudges into the user interface. These aims were guided by the revised Observational Research Behavioral Information Technology model. A total of 50 clinicians who used the original version of the tool were surveyed with a quantitative instrument that we developed based on a behavior theory framework—the Capability-Opportunity-Motivation-Behavior framework. A semistructured interview guide was developed based on the survey responses. Inductive methods were used to analyze interview session notes and audio recordings from 12 interviews. Revised versions of the tool were developed that incorporated nudges.

**Results:**

Functional prototypes were developed by using Axure PRO (Axure Software Solutions) software and usability tested with end users in an iterative agile process (n=10). The tool was redesigned to address 4 identified major barriers to tool use; we included 2 nudges and a default. The 6-month pilot trial for the tool was launched on October 1, 2021.

**Conclusions:**

Clinicians highlighted several important psychological and behavioral barriers to CDS use. Addressing these barriers, along with conducting traditional usability testing, facilitated the development of a tool with greater potential to transform clinical care. The tool will be tested in a prospective pilot trial.

**International Registered Report Identifier (IRRID):**

DERR1-10.2196/42653

## Introduction

### Background

US citizens receive only half of the recommended medical care [1], and one-third of the care received is unnecessary [2]. The disparity between usual and evidence-based clinical practice is the reason behind one-third of hospital deaths [3] and results in an estimated loss of US $380 billion each year [4]. Computerized clinical decision support (CDS) attempts to close this gap by bringing meaningful and relevant evidence to health care providers at the point of decision-making. Health care providers, policy makers, experts, and consumers have identified CDS tools as key tools for revolutionizing health care [5-7]. However, the moderate improvements in care resulting from CDS [8-14] have been significantly limited by consistently low health care provider adoption [15,16]. There is a critical need to identify strategies for increasing health care providers’ adoption of CDS.

Health care provider attitudes toward CDS, specifically psychological and behavioral barriers, are not typically addressed during any stage of CDS development, although they represent an important barrier to adoption [17]. Psychological and behavioral barriers to use must be addressed along with traditional barriers to usability to improve adoption and, as a result, improve clinical impact. Our study’s objective is to employ a user-centered design process to develop a CDS tool—the pulmonary embolism (PE) risk calculator (PERK)—for PE risk stratification in the emergency department that incorporates a behavior theory–informed nudge to address identified behavioral barriers to use.

### PE Risk Stratification

The use of PE risk stratification tools in the emergency department setting can reduce unnecessary computed tomography (CT) scans [18,19]. The 2-tiered *Wells score for PE* clinical prediction rule classifies patients as those with a low or high probability of PE based on key elements of their histories and physical exams. This rule recommends the use of a laboratory value—the dimerized plasmin fragment D (D-dimer) assay—to rule out PE in low-risk patients. This eliminates the need for imaging in patients with a low probability of PE and a negative D-dimer assay result. The use of clinical prediction rules to assess pretest probability before performing a CT scan reduces testing by 25%, without any missed PEs [18,19]. This reduction in CT scan use is important, as each test carries a 14% risk of contrast-induced nephropathy [20] and a lifetime malignancy risk that can be as high as 2.76% [21]. Additionally, incidental findings requiring diagnostic follow-up are found in 24% of tests, increasing both the costs and the harms from repeat imaging [22].

### Nudges and Behavioral Economics

The field of behavioral economics studies the effects of psychological, social, cognitive, and emotional factors on the economic decisions of individuals. This field is contributing to a growing body of research demonstrating the usefulness of addressing subtle attitudes, beliefs, and habits that result in individuals behaving irrationally [23,24]. In behavioral economics, nudges are used to influence behavior. *Nudges* are defined as positive reinforcement and indirect suggestions that have a nonforced effect on decision-making [25]. For example, the default options for organ donation consent result in striking differences in enrollment [26]. An example in health care is comparing health care providers to peers, which has been effective at reducing antibiotic prescribing for upper respiratory infections [27].

Nudges have been successful in influencing health care provider decision-making. There is a growing body of evidence demonstrating that health care provider decisions are also subject to variability resulting from psychological and emotional factors [28-30]. A recent meta-analysis found that direct education approaches did not result in sustained reductions in inappropriate antibiotic prescriptions [31]. However, a large randomized controlled trial found that a social comparison nudge decreased the rate of health care provider antibiotic prescribing for upper respiratory infections from 19.9% to 3.7% [27]. These results were durable at 3 months [32]. Integrating behavioral economics strategies into electronic health records (EHRs) by using CDS tools is a novel approach to improving guideline adherence that also seeks to minimize negative impacts on clinical workflows and cognitive loads.

### The Observational Research Behavioral Information Technology Model for Behavioral Intervention Development

Our developmental work was guided by the revised Observational Research Behavioral Information Technology (ORBIT) model, which provides a tested framework for behavioral intervention development [33]. The model includes several phases with well-defined outcomes and milestones, culminating in the testing of the intervention in a large-scale effectiveness trial. The model features several bidirectional arrows, encouraging iterative development and refinement. The use of this model to support the development of behavioral interventions is key to ensuring that scientific developments contribute to the development of effective interventions. The work described herein details the development of our intervention (ie, Phase 1 Design).

## Methods

Our study used a user-centered and behavior theory–based approach to achieve the following two aims: (1) use mixed methods to identify health care provider barriers to the use of an active CDS tool for PE risk stratification and (2) develop a new CDS tool—the PERK—that addresses behavioral barriers to health care providers’ adoption of CDS by incorporating nudges into the user interface. These aims were guided by the revised ORBIT model.

### Ethics Approval

All study activities took place at a large academic health system in the New York City metropolitan area and were approved by the Northwell Health Institutional Review Board (IRB# 18-0714).

### Aim 1: Identification of Barriers to CDS Tool Adoption (Discovery)

#### Original CDS Tool for PE Risk Stratification

We previously developed a CDS tool for PE risk stratification in the emergency department, based on the Wells criteria for PE*,* that was found to be effective when used, with a 37% reduction in unnecessary CT scan use among adopters [34]. However, it was only used by 15% of health care providers. Low health care provider adoption significantly limited the impact of the tool on overall health care provider behavior. The original tool was active at 2 large academic emergency departments at the time of the development of the new tool, that is, emergency departments at the same institution where our study took place.

Additional details about the design, implementation, and evaluation of the original tool are available in a previous publication [34]. Briefly, emergency clinicians entering any electronic order for the diagnosis of PE (D-dimer testing, ventilation/perfusion scan, or CT scan) are routed to the tool if they answer “yes” to a dialog box asking “Are you considering PE?” The tool functions as an expanded order set that allows clinicians to formally calculate the pretest probability of PE, using the 3-tiered Wells criteria for PE. For low-risk patients, the tool only allows clinicians to order D-dimer laboratory testing, and for patients with an intermediate or high risk of PE, it allows for the ordering of D-dimer testing, ventilation/perfusion scans, or CT scans. At any time however, the tool can be dismissed by clinicians. The tool was developed by using adaptive principles of web and health information technology design, which are detailed in several previous publications [35-38].

#### Quantitative Assessment of Barriers to CDS Adoption

**Figure 1:**
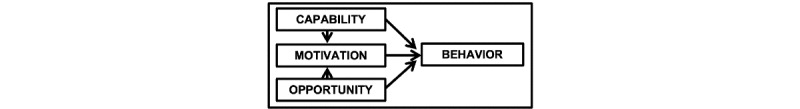
The Capability-Opportunity-Motivation-Behavior model.

To determine the reasons for the low rate of health care provider adoption (15%), we surveyed 50 users (emergency department residents and attending physicians) of the original tool with a quantitative instrument that we developed based on the Capability-Opportunity-Motivation-Behavior (COM-B) behavior theory framework. The COM-B framework specifies that changing behavior requires changing capabilities, opportunities, and/or motivations (Figure 1) [39]. It divides *capability*, *opportunity*, and *motivation* into specific subcategories to allow for a detailed assessment of barriers in each category. These are further broken down into theoretical domain functions. The COM-B framework is intended to be comprehensive, parsimonious, and applicable to all behaviors, and it was developed based on existing theories of behavior at a US consensus meeting of behavioral theorists [39]. Our survey assessed the prevalence of 12 behavioral barriers. These 12 behavioral barriers were selected from the theoretical domain functions based on their relevancy to CDS use. Users identified 4 barriers as primary barriers to adoption; they were reported by greater than 70% (35/50) of participants. Almost all (91%, 45/50) of the users who reported low social opportunity also reported not having the intention to use the tool (Table 1).

**Table 1:** Major barriers to clinical decision support tool use identified by the survey (N=50).

Example quotes	Capability-Opportunity-Motivation-Behavior domain	Respondents reporting the same barrier, n (%)
“my colleagues don’t use the tool”	Low social opportunity (cultural norms)	35 (70)
“good doctors don’t need the tool”	Low reflective motivation (professional identity)	39 (78)
“when I see the tool pop up I feel annoyed”	Low automatic motivation (emotion)	43 (86)
“the EMR has too many alerts to address”	Low psychological capability (attention)	45 (91)

#### Qualitative Assessment of Barriers to CDS Adoption

Based on the survey results and the COM-B framework, we developed a semistructured interview guide to further our understanding of behavioral barriers to tool use. Thematic saturation was reached after the 12th interview, with no new insights obtained by the 12th participant’s interview. Inductive methods were used to analyze session notes and audio recordings, using the COM-B framework as a guiding theory. Interviews were conducted with 12 health care providers, (emergency department residents, attending physicians, and physician assistants). The interviews were 30 to 60 minutes long and were conducted by study personnel. Complete study methods and results are available in a previous publication [40].

The following six major barriers were identified: (1) *Bayesian reasoning*, (2) *fear of missing PEs*, (3) *time pressure/cognitive load*, (4) *gestalt includes the Wells criteria for PE*, (5) *missed risk factors*, and (6) *social pressure* (Table 2). Clinicians highlighted the belief that the tool was not useful to them because all elements of the Wells criteria for PE were incorporated into their gestalt. The *fear of missing PEs* was another major theme. Clinicians felt worried about department quality improvement reviews or legal action. *Time pressure/cognitive load* was also highlighted as a major barrier to tool use. Although clinicians denied that cognitive loads kept them from using the tool, many clinicians spontaneously mentioned that they preferred the PE rule-out criteria due to their simplicity. The PE rule-out criteria are validated for use to rule out PE in low-risk patients if 8 criteria are not met [41].

**Table 2:** Major barriers to clinical decision support tool use refined by the semistructured interviews (n=12; adapted from *Barriers to the Use of Clinical Decision Support for the Evaluation of Pulmonary Embolism: Qualitative Interview Study* by Richardson et al [40]).

Theme	Quotes
*Bayesian reasoning*	“I don’t think [pretest probability] matters for the CT scan.... I’ve been told if you order a CT, you’ll either see it or you won’t.”
*Fear of missing pulmonary embolisms*	“…the environment with [quality improvement oversight] and the medical-legal situation, I might argue the threshold to test here is 0%.”
*Time pressure/cognitive load*	“the biggest takeaway that you could take from interviewing ER providers is time, like that’s the thing that matters most to us. Time and like ease of use.”
*Gestalt includes the Wells criteria for pulmonary embolism*	“I never use [the clinical decision support tool], I have done the scoring in my head.”
*Missed risk factors*	“my clinical gestalt has red flags for things that are not on Wells’....it doesn’t have some of the younger woman risk factors like OCPs and smoking history”
*Social pressure*	“it does happen once in a while that I’ll think this person, the patient, can get away with a D-dimer alone but the [physician assistant] or the learner wants to do a CT Scan, and I’m not averse to letting that go”

Additional themes included *Bayesian reasoning*, *missed risk factors*, and *social pressure*. The *Bayesian reasoning* theme reflected some clinicians not recalling that the posttest probability of PE would be impacted by the pretest probability of PE, which is predicted by the CDS tool, regardless of the results of the CT scan. The *missed risk factors* theme reflected clinicians’ mistrust of the CDS tool, as the Wells criteria for PE do not explicitly include a few known risk factors for PE. The *social pressure* theme reflected many clinicians reporting that other members of the care team, including patients and their primary care physicians, could influence their decision to not use or not follow the recommendations of the tool.

#### Clinical Workflow Analysis

To discover clinical workflow barriers to CDS tool use that might not be provided by users during interview sessions, we shadowed 3 emergency department attending physicians for 1 to 2 hours each. The workflow analysis was adapted from the Agency for Healthcare Research and Quality recommendations on workflow assessments [42]. Direct observation sessions highlighted the significance of the barrier *time pressure/cognitive load* in the analysis of interview themes. All health care providers shadowed demonstrated high patient loads, task complexity, and task fragmentation. Health care providers juggled multiple complex patients simultaneously with constant interruptions. The clinical workflow steps were consistent with the reports that were detailed during the interviews for our study and the formative work for the development of the original tool. Health care providers typically reviewed the emergency department triage notes for patients’ initial vitals and chief complaints. Afterward, they saw patients in their room to perform a history and physical examination. Finally, they placed orders in the EHR.

### Aim 2: Develop a New CDS Tool—the PERK (Design—Phases 1A and 1B)

#### Development of Potential Nudges

Based on the survey results, semistructured interviews, and clinical workflow analysis, wireframes of 6 potential nudges were developed. Each potential nudge wireframe was designed to target an identified barrier to tool use and was informed by a COM-B category and subcategory (theoretical domain; Table 3). Wireframes are nonfunctional depictions of the user interface. Wireframes were reviewed and revised during a design thinking workshop and then developed into functional tool prototypes.

**Table 3:** Major barriers to clinical decision support use, Capability-Opportunity-Motivation-Behavior (COM-B) categories, theoretical domains, and potential nudges.

Barriers	COM-B categories	Theoretical domain	Potential nudge
*Bayesian reasoning*	Capabilities, psychological	Knowledge	Quiz addressing the false-positive rate of computed tomography scans for low-risk patients
*Fear of missing pulmonary embolisms*	Motivation, reflective	Beliefs about consequences	Text communicating the health system’s support of tool use
*Time pressure/cognitive load*	Capabilities, psychological	Memory, attention, and decision processes	Estimated time to completion (12 seconds)
*Gestalt includes the Wells criteria for pulmonary embolism*	Motivation, reflective	Personal identity	Peer comparison (user compared to tool adopter)
*Missed risk factors*	Capabilities, psychological	Knowledge	List of conditions for showing when it is appropriate and inappropriate to use the tool
*Social pressure*	Opportunity, social	Social influences	Peer comparison (user compared to department mean)

#### Design Thinking Workshop

The results of the survey and semistructured interviews, the refined identified barriers to tool use, and the results of the workflow analysis were presented to several experts in health informatics, usability testing, behavioral science, and health services research and emergency department clinicians during a formal design thinking workshop. The session was guided by a user-centered design facilitation protocol that was used to lead the group through a presentation about behavioral economics concepts, CDS, and the emergency department clinical workflow before an idea generation exercise. Barriers were presented first, and participants were asked to create their own potential nudges for increasing health care provider adoption. Attendees worked in groups and submitted answers by using Post-it notes. Groups presented their ideas in a sharing and discussion session. Later wireframes of potential nudges were presented to the group for feedback. Insights from this workshop were used to further revise the potential nudges and build the tool prototypes.

## Results

This section focuses on the initial testing of the decision support tool and how feedback from each phase of testing further influenced the content and design of the tool. The 6-month pilot trial for the tool was launched on October 1, 2021.

### Usability Testing

CDS tool prototypes were built, using the prototype development software Axure RP 8 PRO (Axure Software Solutions). Think-aloud usability testing was conducted by using these prototypes with 6 health care providers (emergency department residents, attending physicians, and physician assistants). Prototypes are functional representations of the final user interface that mimic the full user experience. During think-aloud usability testing sessions, participants verbalized their thought processes while interacting with the tool prototype [43]. They were asked to complete the specific steps necessary for using the tool. They were given written clinical scenarios involving low-, intermediate-, or high-probability PE cases.

Following the usability testing sessions, the potential nudges were revised based on participant feedback. The layout, text, colors, and placement of each element of the CDS tool were all iteratively revised to facilitate ease of use and perceived usefulness. Several nudges were eliminated, and 2 were selected to be included in the final design. The nudge directed at the *Bayesian reasoning* barrier was eliminated based on feedback that health care providers would not take the time to respond to even a 1-question quiz during a patient care session. The nudge directed at the *fear of missing PEs* barrier was revised so that the language was clearer, more directed, and insertable into health care providers’ notes. The nudge directed at the *time pressure/cognitive load* barrier was eliminated based on feedback that no estimated length of time for tool completion could be short enough to be perceived positively.

The nudge directed at the *gestalt includes the Wells criteria for PE* barrier was eliminated based on feedback from the health care providers that they did not see the relevance of comparing themselves to physicians outside of their department who might care for different patient populations. The nudge directed at *social pressure* was revised several times to address health care provider questions about how the data were calculated and to facilitate fast understanding. The final user interface was built to address the *fear of missing PEs*, *gestalt includes the Wells criteria for PE*, *time pressure/cognitive load*, and *social pressure* barriers. To address major concerns about *time pressure/cognitive load*, we developed an automated method for calculating the Wells criteria for PE, as well as a tool activation and suppression algorithm to reduce alert fatigue. A default was added to the user interface; recommended orders are highlighted in green, and 1 click is needed to proceed to ordering. Orders that are not recommended turn yellow if they are clicked once and are ordered if they are clicked a second time.

**Figure 2:**
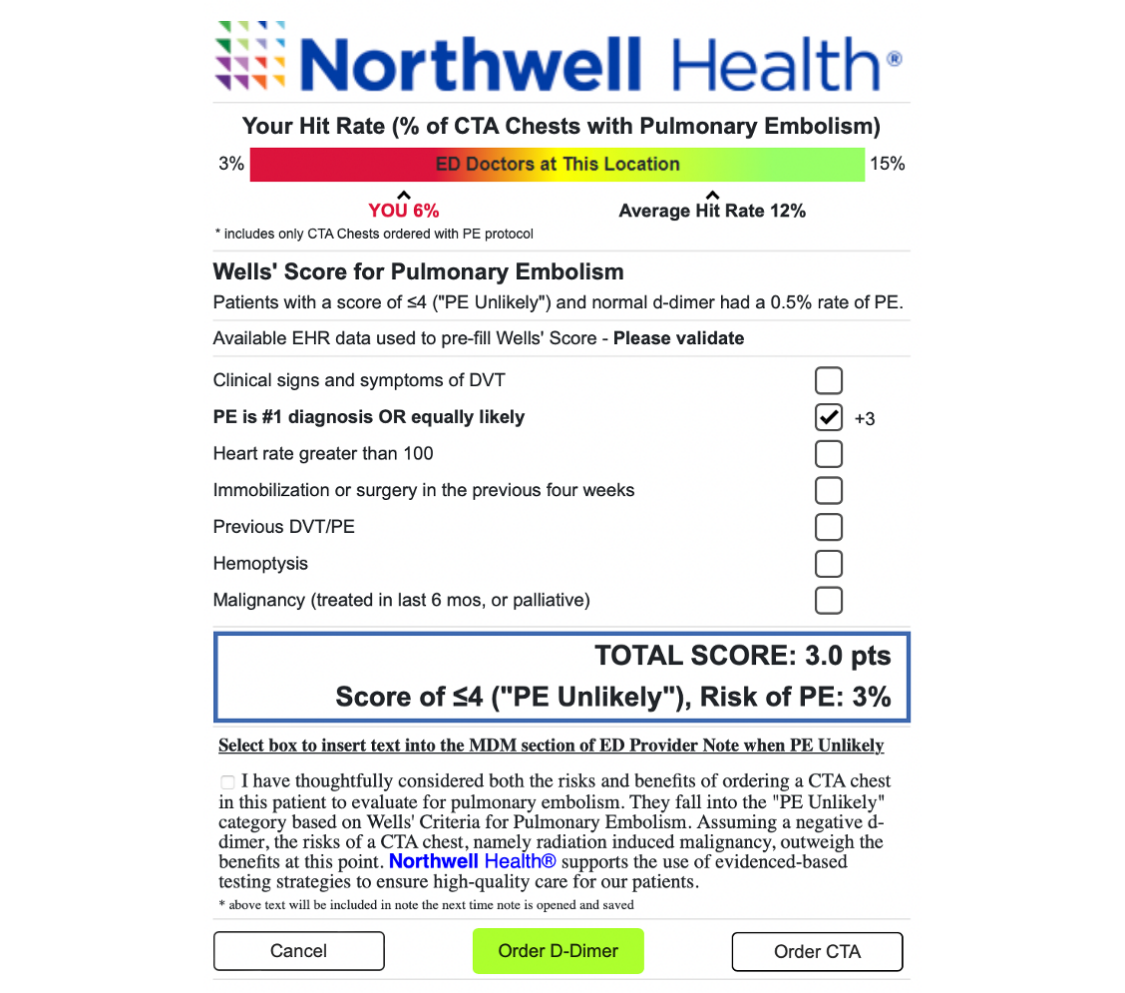
The pulmonary embolism risk calculator tool user interface.

After iterative revision, the final PERK tool user interface was developed, as shown in Figure 2.

### Automatic Calculation of the Wells Criteria for PE

To address the major barrier *time pressure/cognitive load*, we designed an automated process by using data elements from the EHR and health information exchange to fill in information for each Wells criterion. A detailed description of the methodology for and results of the validation study are presented elsewhere [44]. Briefly, we used a combinatorial keyword search method to query free-text chief complaint and chief complaint quote fields for the criteria “clinical signs and symptoms of DVT” and “hemoptysis.” We used diagnoses listed in past medical histories for the “history of PE/DVT,” “malignancy,” and “immobilization or surgery in the previous 4 weeks” criteria. The presence of brief operative notes, including those regarding general anesthesia, was also used to identify patients who underwent recent surgery for the “immobilization or surgery in the previous 4 weeks” criterion. Vital signs taken during an encounter were used for the “heart rate greater than 100” criterion. All patients were given 3 points for the subjective criterion “an alternative diagnosis is less likely than PE” if a CT scan was ordered for the patients to evaluate for PE.

To validate the automated process, automatically calculated Wells scores were compared to those derived from a 2-clinician chart review. Patients classified as “PE likely” by the automated process (126/202, 62.4%) had a PE prevalence of 15.9% (20/126), whereas those classified as “PE unlikely” (76/202, 37.6%) had a PE prevalence of 7.9% (6/76). With respect to the classification of the patients as “PE likely,” the automated process achieved an accuracy of 92.1% when compared with the chart review. This methodology was used to automatically calculate patients’ Wells criteria for PE when the tool was triggered, as well as to prefill information for each criterion when the CDS was displayed to health care providers.

### Tool Triggering and Suppression Algorithm

An algorithm was created to dictate the rules for triggering and suppressing the CDS tool. Our goals were to integrate the tool into the current clinical workflow for patients with suspected PE and to suppress the tool in cases where it was not likely to be helpful. The final rules for triggering and suppression were as follows. First, an assessment is triggered based on the placement of a “CTA Chest PE Protocol w/ IV Contrast” (CT scan) order. This assessment considers the automatically calculated Wells criteria for PE and any orders that were placed for a D-dimer laboratory test during the patient encounter. Second, the tool is suppressed for patients with a Wells score >4 and/or if any order has been placed for a D-dimer laboratory test during the patient encounter. The tool is not suppressed for patients with a Wells score of 4 for whom a D-dimer test was not ordered.

Patients with a Wells score of >4 and/or a positive D-dimer test result are classified as “PE Likely” and should undergo a CT scan without interruption. Health care providers placing an order for a CT scan for patients with a Wells score 4 for whom a D-dimer test was not ordered are interrupted by the tool, as shown in Figure 1. The decision was made to suppress the tool for patients for whom a D-dimer test was not ordered, as opposed to suppressing the tool only for patients for whom the laboratory test was ordered and showed abnormal results, as the tool primarily attempts to prompt health care providers intending to order a CT scan for low-risk patients to instead place an order for a D-dimer test. If this has already been done and the health care provider has decided to not wait for the results or to place a CT scan for a low-risk patient with a normal D-dimer test result, then suggesting that they order a D-dimer test would not be likely to effectively influence their decision-making. Our goal was to minimize alert fatigue by increasing the efficiency of the tool.

## Discussion

We describe the development of a CDS tool for PE risk stratification in the emergency department setting that incorporates behavior theory–informed nudges to address barriers to health care provider use. Behavioral barriers are not typically addressed during any stage of CDS development, although they represent an important barrier to adoption. We employed user-centered design principles and were guided by the ORBIT model, which advocates for an iterative development approach. Our developmental work included a mixed methods assessment of barriers to tool use and the frequent usability testing of functional prototypes. The COM-B behavioral framework guided the assessment of barriers to tool use. The final user interface included 2 nudges, in addition to a default, and addressed 4 of the 6 identified major barriers to tool use.

Our use of one of the tools of behavioral economics—nudges—is an innovative way to address common psychological, behavioral, and environmental barriers to CDS use. The development of the user interface of the PERK served as a key step and demonstrated that nudges can be incorporated into CDS. The next step is to establish the effectiveness of nudges in clinical practice. Such an evaluation is currently underway, with results expected in the coming months. If, as expected, we find that the adoption of the tool increases, we will conduct studies to evaluate which nudge works best, whom the nudges work best for, and when the nudges work best. These subsequent studies will be conducted by using the multiphase optimization strategy and will allow for the identification of the most effective intervention strategies [45]. This will pave the way for more tailored CDS tools.

There are several strengths and limitations to our approach. The strengths include the fact that the tool was designed for the emergency department clinical setting. The emergency department is one of the more difficult clinical settings to design CDS tools for, due to the high-pace, urgent, and fragmented nature of clinical care. The limitations include the single-center nature of our work. We included several types of emergency department health care providers from 2 hospitals in the same large academic medical center. Therefore, our results may not apply to other hospital systems.

Incorporating principles from behavioral economics into the development of CDS tools has shown increasing promise as a strategy for improving tool effectiveness while minimizing health care provider time and cognitive load burdens. Others have used principles of behavioral economics to influence health care provider behavior through asynchronous peer comparison emails, posters, and the grouping of treatment options [27,29,32,46]. Our work serves as a proof of principle for using this novel approach for CDS development.

## Appendix

Multimedia Appendix 1 Peer-review report.

## Abbreviations

CDS: clinical decision support
COM-B: Capability-Opportunity-Motivation-Behavior
CT: computed tomography
D-dimer: dimerized plasmin fragment D
EHR: electronic health record
ORBIT: Observational Research Behavioral Information Technology
PE: pulmonary embolism
PERK: pulmonary embolism risk calculator

## Data Availability

The data that support the findings of our study are not publicly available due to restrictions, as making them publicly available could compromise the privacy of the research participants. The data restrictions are being imposed by Northwell Health.
