# Supplementary material for: Nudging Health Care Providers’ Adoption of Clinical Decision Support: Protocol for the User-Centered Development of a Behavioral Economics–Inspired Electronic Health Record Tool
Source: JMIR Res Protoc. 2023 Jan 18;12:e42653. doi: 10.2196/42653 (PMC9892982; doi:10.2196/42653)
Supplement: Multimedia Appendix 1 [file resprot_v12i1e42653_app1.pdf]

SUMMARY STATEMENT
( Privileged Communication )

**PROGRAM CONTACT:**
Sandra Colombini-Hatch
301-435-0222
hatchs@nhlbi.nih.gov

*Release Date:* 08/10/2018
*Revised Date:*

*Application Number:* 1 K23 HL145114-01

Principal Investigator

RICHARDSON, SAFIYA

Applicant Organization: FEINSTEIN INSTITUTE FOR MEDICAL RESEARCH

*Review Group:* MPOR (OA)
NHLBI Mentored Patient-Oriented Research Review Committee

*Meeting Date:* 06/21/2018
*Council:* OCT 2018
*Requested Start:* 09/01/2018
*RFA/PA:* PA18-374
*PCC:* LLLJAN

*Project Title:* Nudging Provider Adoption of Clinical Decision Support

*SRG Action:* Impact Score:20
*Next Steps:* Visit https://grants.nih.gov/grants/next_steps.htm
Human Subjects: 30-Human subjects involved - Certified, no SRG concerns
Animal Subjects: 10-No live vertebrate animals involved for competing appl.
Gender: 1A-Both genders, scientifically acceptable
Minority: 1A-Minorities and non-minorities, scientifically acceptable
Children: 3A-No children included, scientifically acceptable

| Project Year | Direct Costs Requested | Estimated Total Cost |
|---|---|---|
| 1 | 158,234 | 170,893 |
| 2 | 160,801 | 173,665 |
| 3 | 160,903 | 173,775 |
| 4 | 160,950 | 173,826 |
| 5 | 160,599 | 173,447 |
| TOTAL | 801,487 | 865,606 |

ADMINISTRATIVE BUDGET NOTE: The budget shown is the requested budget and has not been adjusted to reflect any recommendations made by reviewers. If an award is planned, the costs will be calculated by Institute grants management staff based on the recommendations outlined below in the COMMITTEE BUDGET RECOMMENDATIONS section.

**1 K23 HL 145114 - 01 RICHARDSON, SAFIYA**

**RESUME AND SUMMARY OF DISCUSSION:** This is a new K23 application from Dr. Safiya Richardson in which she has proposed a research career development plan focused on the development and testing of a computerized clinical decision support for pulmonary embolism risk prediction. The Candidate is exceptional. She has five first authored publications and strong letters of support. She has significant research experience in developing health information technologies to assist provider decision making. The Mentors are outstanding, have the necessary expertise, and have established relationships with the Candidate. The Research Plan seeks to improve "evidence-based" care at the point of decision making by using behavioral "nudging" to increase adoption of clinical decision support tools. The research is based on a strong premise and potentially impactful; however, there was no discussion of alternative strategies. Overall, this is an outstanding application from an exceptional young investigator. Reviewer enthusiasm was high despite some weaknesses in the research strategy.

**DESCRIPTION (provided by applicant):** With the long-term career goal of becoming a leading independent researcher transforming health information technology, Safiya I. Richardson, MD, MPH, proposes a mentored research project and specific career development plan which will prepare her to use behavioral science to develop effective clinical decision support (CDS). The disparity between usual and evidenced based clinical practice is responsible for a third of hospital deaths and waste estimated at 380 billon dollars each year. Computerized CDS has the potential to narrow this gap by bringing meaningful and relevant evidence to health care providers at the point of decision making. However, moderate improvements in care seen with CDS are significantly limited by consistently low provider adoption, estimated at 10%. This project uses behavioral theory and key principles of behavioral economics to illuminate and address barriers to provider adoption of CDS. Using the Capability Opportunity Motivation Behavior (COM-B) framework Dr. Richardson will examine barriers to provider adoption of a pulmonary embolism risk prediction CDS tool. The use of CDS to assess pre-test probability before computed tomography pulmonary angiography reduces testing by 25% without any missed pulmonary emboli. Routine use by providers would result in 600,000 fewer scans, 84,000 fewer cases of contrast induced nephropathy and prevent 3,000 malignancies as well as 2,000 cancer deaths in the United States every year. The overall objective of this training application is to develop and evaluate the feasibility and preliminary efficacy on provider adoption of a new tool that incorporates nudges designed to address barriers to tool use. Nudges are applications of behavioral science used by behavioral economists, defined as positive reinforcement and indirect suggestions which have a non-forced effect on decision making. The proposal aims to: 1) develop nudges designed to address identified behavioral barriers to adoption, 2) build and conduct iterative usability testing on prototypes of the new tool, 3) evaluate the feasibility and preliminary efficacy on provider adoption of the new tool compared to the current tool, in a pilot trial. This project uses a multistage mixed methods framework. It is the first to evaluate the impact of nudges on provider adoption of CDS. This research is complemented by career development activities, including formal training in health informatics, behavioral science, mixed methods and clinical trial design. With the guidance of an experienced mentoring team, the proposed research and training activities will lead to the development a competitive R01 grant application to assess the effectiveness of the new tool to improve health outcomes.

**PUBLIC HEALTH RELEVANCE:**
Computerized clinical decision support attempts to close the gap between usual and evidence based clinical practice by bringing relevant evidence to health care providers at the point of decision making, however, the moderate improvements in care seen with these tools have been significantly limited by consistently low provider adoption. This proposed study aims to use the principles of behavioral economics (i.e. the "nudge") in the development of a pulmonary embolism risk prediction clinical

decision support tool to improve provider adoption. Increasing the adoption of clinical decision support tools will improve quality of care, decrease morbidity, mortality and health care waste.

**CRITIQUE 1:**

Candidate: 1
Career Development Plan/Career Goals /Plan to Provide Mentoring: 3
Research Plan: 3
Mentor(s), Co-Mentor(s), Consultant(s), Collaborator(s): 2
Environment Commitment to the Candidate: 1

**Overall Impact:**
Dr Richardson is a clinician scientist in her second year as junior faculty. She is working in the field of implementation science to understand the behavioral reasons why providers don't utilize clinical decision support. Much of her underlying work has focused on both the usability testing and the quantitative survey data surrounding a lack of adoption for a CDS tool to reduce the number of CTs for PE rule out.

The proposed training plan is integrated deeply with the research plan and includes a certificate in clinical informatics; mixed methods/ qualitative and behavioral economics and research methods and clinical trials. The research aims seek to develop nudges to address the identified barriers to CDS adoption (semi structured interviews done for low inter and high tool users). To do iterative testing on the prototypes of the new tool using traditional usability testing (Think aloud and near live). The third aim is testing the new tool in a pre-post design at 2 hospitals among 150 providers for acceptability adoption/ uptake and satisfaction.

This is the aim that is methodologically the weakest and could be strengthened if using a difference in difference design or even doing a small cluster RCT at the 2 sites with the provider as the unit of randomization. This would likely be feasible during the K time because of the automated nature in flipping on a CDS tool at the provider level and the big data collection using informatics to collect all clinical outcomes at the patient level for those seen with chest pain or SOB etc.

**1. Candidate:**
**Strengths**
- Fantastic candidate with clear career trajectory and dedication to improving clinical care.
- She has had foundational research training and experiential learning through the NIH supplement.
- The reduction of unnecessary testing is an important concept which is often more difficult to change in providers than asking them to perform additional tests or add a behavior to meet a quality metric. The use of behavioral economics to understand these concepts would make Dr Richardson a leader in this field and her work would contribute to this understanding.

**Weaknesses**
- None noted.

**2. Career Development Plan/Career Goals & Objectives:**
**Strengths**
- Good collaborative mentorship team who have a record of working with Dr Richardson.
- There is a clear plan for learning and advancement, although didactics are heavy in years 1-3.

**Weaknesses**
- I am uncertain of the time commitment need for the completion of the Certification program in Health IT. I can see how it may be beneficial; however, it appears to be a big portion of time in

year 2 when much of the research will also be starting up and underway. The table notes only about 5-6 hours a week but that seems unlikely for a formal certificate program.

**3. Research Plan:**
**Strengths**
- CDS is an important tool for health systems to remain agile and increase their ability to apply standardized and evidenced based knowledge at the frontline of patient care.
- The understanding of the behavioral approaches to adoption and implementation/uptake of the CDS is of paramount importance to its success.
- The use of mixed methods to iterate the CDS is a strength and would add to Dr Richardson's skills.

**Weaknesses**
- The efficacy trial to assess adoption of the PERK tool compared to standard CDS without the behavioral nudge is a strength; however, the pre-post design is not optimal. Consider a difference in difference design if there are enough timepoints pre and post; or consider randomizing at the level of the provider.

**4. Mentor(s), Co-Mentor(s), Consultant(s), Collaborator(s):**
**Strengths**
- Great team with complementary expertise who have clearly been working with Dr. Richardson and advising her during the course of her residency, fellowship and as a junior faculty.

**Weaknesses**
- None

**5. Environment and Institutional Commitment to the Candidate:**
**Strengths**
- Strong institutional support as well as enrollment in the program for junior faculty clinician scientists (Health Outcomes research career development program).

**Weaknesses**
- None noted.

**Study Timeline:**
**Strengths**
- Given the duration of the follow-up after tool roll out, it can easily be deployed as a pragmatic trial with randomization at the provider level. This can help with understanding both the secular trends and the effect of the tool.

**Weaknesses**
- None

**CRITIQUE 2:**

Candidate: 1
Career Development Plan/Career Goals /Plan to Provide Mentoring: 2
Research Plan: 4
Mentor(s), Co-Mentor(s), Consultant(s), Collaborator(s): 1
Environment Commitment to the Candidate: 1

**Overall Impact:**
This K23 application will provide career development for the applicant through a mentored research project and specific career development plan which will prepare her to use behavioral science to develop effective clinical decision support (**CDS**). The applicant, mentors and environment are outstanding. The overall objective of this application is to develop and evaluate the feasibility and

preliminary efficacy on provider adoption of a new tool that incorporates “nudges” designed to address barriers to tool use. The research plan addresses a significant scientific gap and may inform developments of new methods to increase provider’s adoption of CDS tools; thus, improving quality of care while reducing costs. There are a few minor methodological concerns that do not significantly diminish the enthusiasm for the project. The overall impact is high.

**1. Candidate:**
**Strengths**
- The candidate obtained her medical degree and MPH from Columbia University, New York City, NY, followed by a residency in internal medicine and a fellowship in general internal medicine.
- The candidate is currently a primary care physician and a faculty member at Hofstra Northwell School of Medicine in New York.
- The candidate has significant research experience focusing on developing high impact health information technology to assist provider decision making.
- A current recipient of a Research Supplement to Promote Diversity in Health-Related Research from the NIAID.
- Long-term career goal is to use the principles of behavioral science to develop and implement high impact health information technology and Clinical Decision Support (CDS).
- Published 9 peer-reviewed manuscripts (5 as first author).
- The letters of recommendation are uniformly strong.
- There is evidence of previous collaboration with the mentors.

**Weaknesses**
- None noted.

**2. Career Development Plan/Career Goals & Objectives:**
**Strengths**
- The didactic coursework is tied specifically to proposed research and career goals.
- The training plan includes sufficient detail linking proposed coursework and the contribution of each proposed course to the candidate's career objectives.
- The plan includes appropriate training in the responsible conduct of research.

**Weaknesses**
- Methods of evaluation and training milestones are not sufficiently addressed.

**3. Research Plan:**
**Strengths**
- Computerized clinical decision support (CDS) has the potential to narrow the gap between “usual” and “evidence-based” care at the point of decision making.
- The central hypothesis is that the addition of a “nudge” [a positive reinforcement and indirect suggestions which have a non-forced effect on decision making] designed to address psychological barriers to CDS use will improve provider adoption.
- The project is underpinned by a strong central premise. The study is conceptually innovative, as it proposes to use a behavioral framework (COM-B) to examine barriers to and facilitators of provider adoption of CDS and to evaluate the impact of nudges to increase provider adoption of CDS.
- The first aim is to develop “nudges” designed to address identified behavioral barriers to adoption. The investigator proposes to create wireframes of the new CDS tool and to incorporate four nudges designed to address the identified barriers to tool use into the current tool. The use of semi-structured interviews in groups of varying use rates will provide valuable insight.
- AIM 2 is to build and conduct iterative usability testing on prototypes of the new tool, “PERK”. Iterative usability testing will employ a convergent parallel mixed methods design,

simultaneously collecting both qualitative and quantitative assessments of ease of use. Completion of this aim will allow for finalization of the prototypes and development of the final version of "PERK".

**Weaknesses**

- AIM 3 is to evaluate in a pilot trial the feasibility and preliminary efficacy on provider adoption of the new tool compared to the current tool. However, a higher adoption rate than the current tool may not be meaningful clinically. Is there a specific adoption threshold for a clinically-meaningful difference?
- The success of the research is predicated on *increasing adoption rates* for the new tool. There is insufficient discussion regarding negative findings or alternative approaches.

**4. Mentor(s), Co-Mentor(s), Consultant(s), Collaborator(s):**
**Strengths**

- The primary mentor is Dr. Thomas McGinn. Secondary mentors are: Michael Diefenbach, Ph.D. and Renee Pekmezaris.
- The mentorship team also includes expertise in informatics and biostatistics.
- The roles and responsibilities of each mentor or collaborator are clearly described.

**Weaknesses**

- None noted.

**5. Environment and Institutional Commitment to the Candidate:**
**Strengths**

- Outstanding and conducive to the successful completion of the proposed studies.

**Weaknesses**

- None

**Study Timeline:**
**Strengths**

- Appropriate

**Weaknesses**

- None

**CRITIQUE 3:**

Candidate: 2
Career Development Plan/Career Goals /Plan to Provide Mentoring: 2
Research Plan: 2
Mentor(s), Co-Mentor(s), Consultant(s), Collaborator(s): 2
Environment Commitment to the Candidate: 3

**Overall Impact:**
This is an interesting application by a promising candidate, with a reasonable track record of success, who makes a compelling case for a pivot. After a basis in epidemiology, they propose a pivot to a cleverly combined program of informatics to do clinical decision support and behavioral economics (lightly) to help inform making that decision support actually useful. This seems like a highly productive union.

The program itself is an effort to design a clinical decision support program to reduce low yield CT scans for PE an important goal, and a reasonable initial model system. The Specific Aims of the grant as presented are a little frustratingly un-specific, but when one digs in, one can see a thoughtful build up. In some places greater depth of the motivating behavioral social theory and the link to intervention

selection is desired, but presumably those are what the training program and Dr. Diefenbach's co-mentorship are for. In order to successfully compete for an R01, a much more nuanced grasp of specific behavioral/social theories will be needed, and the intervention choice will need to be rooted in more than the plausibility arguments presented here but the proposal gives reasonable confidence that such growth will occur to offer a promising research career.

**THE FOLLOWING SECTIONS WERE PREPARED BY THE SCIENTIFIC REVIEW OFFICER TO SUMMARIZE THE OUTCOME OF DISCUSSIONS OF THE REVIEW COMMITTEE, OR REVIEWERS' WRITTEN CRITIQUES, ON THE FOLLOWING ISSUES:**

**PROTECTION OF HUMAN SUBJECTS (RESUME): ACCEPTABLE**

**INCLUSION OF WOMEN PLAN (RESUME): ACCEPTABLE**

**INCLUSION OF MINORITIES PLAN (RESUME): ACCEPTABLE**

**INCLUSION OF CHILDREN PLAN (RESUME): ACCEPTABLE; NO CHILDREN; SCIENTIFICALLY JUSTIFIED**

**TRAINING IN THE RESPONSIBLE CONDUCT OF RESEARCH: ACCEPTABLE**

**RESOURCE SHARING PLANS: ACCEPTABLE**

**AUTHENTICATION OF KEY BIOLOGICAL AND/OR CHEMICAL RESOURCES: NOT APPLICABLE (NO RELEVANT RESOURCES)**

**COMMITTEE BUDGET RECOMMENDATIONS: RECOMMENDED AS REQUESTED**

---

Footnotes for 1 K23 HL145114-01; PI Name: Richardson, Safiya

NIH has modified its policy regarding the receipt of resubmissions (amended applications). See Guide Notice NOT-OD-14-074 at http://grants.nih.gov/grants/guide/notice-files/NOT-OD-14-074.html. The impact/priority score is calculated after discussion of an application by averaging the overall scores (1-9) given by all voting reviewers on the committee and multiplying by 10. The criterion scores are submitted prior to the meeting by the individual reviewers assigned to an application, and are not discussed specifically at the review meeting or calculated into the overall impact score. Some applications also receive a percentile ranking. For details on the review process, see http://grants.nih.gov/grants/peer_review_process.htm#scoring.

MEETING ROSTER

NHLBI Mentored Patient-Oriented Research Review Committee
Heart, Lung, and Blood Initial Review Group
NATIONAL HEART, LUNG, AND BLOOD INSTITUTE
MPOR (OA)
06/21/2018 - 06/22/2018

Notice of NIH Policy to All Applicants: Meeting rosters are provided for information purposes only. Applicant investigators and institutional officials must not communicate directly with study section members about an application before or after the review. Failure to observe this policy will create a serious breach of integrity in the peer review process, and may lead to actions outlined in NOT-OD-14-073 at https://grants.nih.gov/grants/guide/notice-files/NOT-OD-14-073.html and NOT-OD-15-106 at https://grants.nih.gov/grants/guide/notice-files/NOT-OD-15-106.html, including removal of the application from immediate review.

CHAIRPERSON(S)

MILLER, KAREN K., MD
PROFESSOR OF MEDICINE
NEUROENDOCRINE UNIT
MASSACHUSETTS GENERAL HOSPITAL
HARVARD MEDICAL SCHOOL
BOSTON, MA 02114

MEMBERS

AMIN, RAOUF S., MD
PROFESSOR AND DIRECTOR
DIVISION OF PULMONARY MEDICINE
CINCINNATI CHILDREN’S HOSPITAL MEDICAL CENTER
CINCINNATI, OH 45229

BADR, M. SAFWAN, MD
PROFESSOR AND CHAIRMAN
DIVISION OF PULMONARY, CRITICAL CARE
AND SLEEP MEDICINE
DEPARTMENT OF INTERNAL MEDICINE
WAYNE STATE UNIVERSITY
DETROIT, MI 48201

BANSAL, NISHA, MD *
ASSISTANT PROFESSOR
DIVISION OF NEPHROLOGY
KIDNEY RESEARCH INSTITUTE
UNIVERSITY OF WASHINGTON
SEATTLE, WA 98104

COLLACO, JOSEPH M., MD *
ASSISTANT PROFESSOR
DEPARTMENT OF PEDIATRIC PULMONARY MEDICINE
DIVISION OF SCHOOL OF MEDICINE
JOHNS HOPKINS UNIVERSITY
DAVID M. RUBENSTEIN CHILD HEALTH BUILDING
BALTIMORE, MD 21287

EISEN, HOWARD J., MD
CHIEF
HEART AND VASCULAR INSTITUTE
MILTON S. HERSHEY MEDICAL CENTER
PENNSYLVANIA STATE UNIVERSITY
HERSHEY, PA 17033

FOLTA, SARA, PHD *
ASSOCIATE PROFESSOR
FRIEDMAN SCHOOL OF NUTRITION
SCIENCE AND POLICY
TUFTS UNVIERSITY
BOSTON, MA 02111

IWASHYNA, THEODORE J., PHD, MD
ASSOCIATE PROFESSOR OF INTERNAL MEDICINE
DIVISION OF PULMONARY AND CRITICAL CARE MEDICINE
UNIVERSITY OF MICHIGAN HEALTH SYSTEM
ANN ARBOR, MI 48109

JEEWA, AAMIR, MD *
SECTION HEAD
DIVISION OF CARDIOLOGY
CARDIOMYOPATHY & HEART FUNCTION PROGRAM
THE HOSPITAL FOR SICK KIDS
UNIVERSITY OF TORONTO
TORONTO, ONTARIO, ON
CANADA

LASKIN, BENJAMIN L., MD *
PROFESSOR
DIVISION OF PEDIATRIC NEPHROLOGY
THE KIDNEY TRANSPLANT AND DIALYSIS PROGRAM
THE CHILDREN'S HOSPITAL OF PHILADELPHIA
UNIVERSITY OF PENNSYLVANIA
PHILADELPHIA, PA 19104

LEDERER, DAVID J., MD
ASSOCIATE PROFESSOR
DEPARTMENTS OF MEDICINE AND EPIDEMIOLOGY
COLUMBIA UNIVERSITY MEDICAL CENTER
NEW YORK, NY 10032

MANCUSO, CAROL A., MD
PROFESSOR OF MEDICINE
DEPARTMENT OF INTERNAL MEDICINE
HOSPITAL FOR SPECIAL SURGERY
WEILL CORNELL MEDICAL COLLEGE
NEW YORK, NY 10021

MANWANI, DEEPA G, MBBS *
ASSOCIATE PROFESSOR
DEPARTMENT OF PEDIATRICS
ALBERT EINSTEIN COLLEGE OF MEDICINE
BRONX, NY 10461

MAU, MARJORIE K. LEIMOMI MALA, MD
PROFESSOR
DEPARTMENT OF NATIVE HAWAIIN HEALTH
JOHN A. BURNS SCHOOL OF MEDICINE
UNIVERSITY OF HAWAII AT MANOA
HONOLULU, HI 96813

MIYAMOTO, SHELLEY D., MD *
ASSOCIATE PROFESSOR
DIRECTOR, HEART FAILURE, AND CARDIOMYOPATHY
PROGRAM
THE CHILDREN'S HOSPITAL
UNIVERSITY OF COLORADO AT DENVER
AURORA, CO 80045

MORRIS, ALISON, MD
PROFESSOR
DIVISION OF PULMONARY, ALLERGY
AND CRITICAL CARE MEDICINE
DEPARTMENT OF MEDICINE
UNIVERSITY OF PITTSBURGH
PITTSBURGH, PA 15213

NIETERT, PAUL J., PHD
PROFESSOR OF BIOSTATISTICS
DEPARTMENT OF PUBLIC HEALTH SCIENCES
MEDICAL UNIVERSITY OF SOUTH CAROLINA
CHARLESTON, SC 29425

NOTH, IMRE, MD
PROFESSOR OF MEDICINE
DEPARTMENT OF PULMONARY
AND CRITICAL CARE MEDICINE
THE UNIVERSITY OF CHICAGO
CHICAGO, IL 60637

O'DONNELL, ANNE E., MD
CHIEF AND PROFESSOR
DIVISION OF PULMONARY AND CRITICAL CARE MEDICINE
DEPARTMENT OF MEDICINE
GEORGETOWN UNIVERSITY HOSPITAL
GEORGETOWN UNIVERSITY
WASHINGTON, DC 20007

PRABHU, SUMANTH D., MD
PROFESSOR AND DIRECTOR
MARY G. WATERS CHAIR OF CARDIOVASCULAR MEDICINE
DIRECTOR, DIVISION OF CARDIOVASCULAR DISEASE
UNIVERSITY OF ALABAMA AT BIRMINGHAM
BIRMINGHAM, AL 35294-0006

RAPHAEL, JEAN L, MD, MPH
ASSOCIATE PROFESSOR OF PEDIATRICS
DEPARTMENT OF PEDIATRICS
BAYLOR COLLEGE OF MEDICINE
HOUSTON, TX 77030

ROUMIE, CHRISTIANNE L., MD *
ASSOCIATE PROFESSOR
DEPARTMENT OF INTERNAL MEDICINE AND PEDIATRICS
VANDERBILT UNIVERSITY
NASHVILLE, TN 37212

SAN JOSE ESTEPAR, RAUL, PHD *
ASSOCIATE PROFESSOR OF RADIOLOGY
DEPARTMENT OF RADIOLOGY
BRIGHAM AND WOMEN'S HOSPITAL
BOSTON, MA 02115-0000

SANTANAM, NALINI, PHD, MPH
PROFESSOR
DEPARTMENTS OF PHARMACOLOGY, PHYSIOLOGY
TOXICOLOGY AND CARDIOLOGY
JOAN C. EDWARDS SCHOOL OF MEDICINE
MARSHALL UNIVERSITY
HUNTINGTON, WV 25755

SINHA, SHANTANU, PHD
PROFESSOR
DEPARTMENT OF RADIOLOGY
SCHOOL OF MEDICINE
UNIVERSITY OF CALIFORNIA - SAN DIEGO
SAN DIEGO, CA 92121

SNYDER, EDWARD L., MD *
PROFESSOR AND DIRECTOR
DEPARTMENT OF LABORATORY MEDICINE
AND BLOOD BANK
YALE-NEW HAVEN HOSPITAL
YALE UNIVERSITY
NEW HAVEN, CT 06504

STONE, KATIE L., PHD
SENIOR SCIENTIST
SAN FRANCISCO COORDINATING CENTER
RESEARCH INSTITUTE
CALIFORNIA PACIFIC MEDICAL CENTER
SAN FRANCISCO, CA 94107

ULLRICH, CHRISTINA K., MD *
SENIOR PHYSICIAN
DEPARTMENT OF PEDIATRIC HEMATOLOGY/ONCOLOGY
CANCER AND BLOOD DISORDERS CENTER
DANA-FARBER CANCER INSTITUTE
HARVARD MEDICAL SCHOOL
BOSTON, MA 02215-5450

VAN DAM, ROBERT M., PHD *
ASSOCIATE PROFESSOR
DEPARTMENT OF PHARMACOLOGY
CRUMP INSTITUTE MOLECULAR IMAGING (CIMI)
UNIVERSITY OF CALIFORNIA, LOS ANGELES
GEFFEN SCHOOL OF MEDICINE
LOS ANGELES, CA 90095-1770

WANG, THOMAS J., MD
PROFESSOR AND DIRECTOR
DIVISION OF CARDIOVASCULAR MEDICINE
VANDERBILT UNIVERSITY MEDICAL CENTER
NASHVILLE, TN 37232

ZHANG, MIN, MD, PHD *
PROFESSOR
DEPARTMENT OF STATISTICS
PURDUE UNIVERSITY
WEST LAFAYETTE, IN 47907

## SCIENTIFIC REVIEW OFFICER

WEBB, STEPHANIE JOHNSON, PHD
SCIENTIFIC REVIEW OFFICER
OFFICE OF SCIENTIFIC REVIEW/DERA
NATIONAL HEART, LUNG, AND BLOOD INSTITUTE
BETHESDA, MD 20892

## EXTRAMURAL SUPPORT ASSISTANT

MCCORKLE, ATHENA M
EXTRAMURAL SUPPORT ASSISTANT
OFFICE OF SCIENTIFIC REVIEW/DERA
NATIONAL HEART, LUNG, AND BLOOD INSTITUTE
BETHESDA, MD 20892

## PROGRAM REPRESENTATIVE

BROWN, MARISHKA, PHD
HEALTH SCIENTIST ADMINISTRATOR
DIVISION OF LUNG DISEASE
NATIONAL HEART LUNG, AND BLOOD INSTITUTE
NATIONAL INSTITUTES OF HEALTH
BETHESDA, MD 20814

CAMPO, REBECCA A., PHD
PROGRAM DIRECTOR
DIVISION OF CARDIOVASCULAR SCIENCES
NATIONAL HEART, LUNG AND BLOOD INSTITUTE
BETHESDA, MD 20892

COLOMBINI-HATCH, SANDRA, MD
PROGRAM OFFICER
DIVISION OF LUNG DISEASES
NATIONAL HEART, LUNG, AND BLOOD INSTITUTE
NATIONAL INSTITUTES OF HEALTH
BETHESDA, MD 20892

COOPER, LAWTON S., MD, MPH
HEALTH SCIENTIST ADMINISTRATOR
CLINICAL APPLICATIONS & PREVENTION BRANCH
NATIONAL HEART, LUNG, AND BLOOD INSTITUTE
BETHESDA, MD 29892

HSU, LUCY L., BS
PROGRAM REPRESENTATIVE
EPIDEMIOLOGY BRANCH
NATIONAL HEART, LUNG AND BLOOD INSTITUTE
NATIONAL INSTITUTES OF HEALTH
BETHESDA, MD 20892

HUANG, LI-SHIN, PHD
HEALTH SCIENTIST ADMINISTRATOR
DIVISION OF CARDIOVASCULAR SCIENCES
NATIONAL HEART, LUNG AND BLOOD INSTITUTE
NATIONAL INSTITUTES OF HEALTH
BETHESDA, MD 20837

KALANTARI, ROYA, BS
PROGRAM ANALYST
DIVISION OF LUNG DISEASE (DLD)
NATIONAL INSTITUTE OF HEART, LUNG AND BLOOD
NATIONAL INSTITUTE OF HEALTH
BETHESDA, MD 20892

LUDLAM, SHARI, PHD
PROGRAM OFFICER
NATIONAL HEART, LUNG, AND BLOOD INSTITUTE
NATIONAL INSTITUTES OF HEALTH
BETHESDA, MD 20892

OCHOCINSKA, MARGARET J., PHD
PROGRAM OFFICER
DIVISION OF CARDIOVASCULAR DISEASES
NATIONAL HEART, LUNG, AND BLOOD INSTITUTE
NATIONAL INSTITUTES OF HEALTH
BETHESDA, MD 20892

REDMOND, NICOLE MD
MEDICAL OFFICER
CLINICAL APPLICATIONS AND PREVENTION BRANCH
NATIONAL HEART, LUNG AND BLOOD INSTITUTE
BETHESDA, MD 20817

REINECK, LORA A., MD
MEDICAL OFFICER
DIVISION OF LUNG DISEASES
NATIONAL HEART, LUNG, AND BLOOD INSTITUTE
NATIONAL INSTITUTES OF HEALTH
BETHESDA, MD 20892

SCOTT, JANE, SCD
PROGRAM OFFICIAL
OFFICE OF RESEARCH TRAINING AND CAREER DEVELOPMENT
OFFICE OF THE DIR, DIV OF CARDIOVASCULAR DISEASE
NATIONAL HEART, LUNG AND BLOOD INSTITUTE
BETHESDA, MD 20892

SRINIVAS, POTHUR R., MPH, PHD
HEALTH SCIENTIST ADMINISTRATOR
DIVISION OF CARDIOVASCULAR SCIENCES (DCVS)
NATIONAL HEART, LUNG AND BLOOD INSTITUTE
NATIONAL INSTITUTE OF HEALTH
BETHESDA, MD 20892

STONEY, CATHERINE, PHD
PROGRAM OFFICER
DIVISION OF CARDIOVASCULAR SCIENCES
NATIONAL HEART, LUNG, AND BLOOD INSTITUTE
NATIONAL INSTITUTES OF HEALTH
BETHESDA, MD 20892

TIGNO, XENIA, PHD
PROGRAM DIRECTOR
DIVISION OF LUNG DISEASES
NATIONAL HEART, LUNG AND BLOOD INSTITUTE
NATIONAL INSTITUTES OF HEALTH
BETHESDA, MD 20892

WANG, WAYNE C., PHD
PROGRAM OFFICIAL
DIVISION OF CARDIOVASCULAR SCIENCES
NATIONAL HEART, LUNG, AND BLOOD INSTITUTE
NATIONAL INSTITUTES OF HEALTH
BETHESDA, MD 20892

WERNER, ELLEN M., PHD
PROGRAM DIRECTOR
DIVISION OF BLOOD DISEASES AND RESOURCES
NATIONAL HEART, LUNG, AND BLOOD INSTITUTE
NATIONAL INSTITUTES OF HEALTH
BETHESDA, MD 20892

## GRANTS MANAGEMENT REPRESENTATIVE

RUNDHAUGEN, LYNN M., MS
GRANTS MANAGEMENT SPECIALIST
DERA, OFFICE OF GRANTS MANAGEMENT
NATIONAL HEART, LUNG, AND BLOOD INSTITUTE
NATIONAL INSTITUTES OF HEALTH
BETHESDA, MD 20892-7956

* Temporary Member. For grant applications, temporary members may participate in the entire meeting or may review only selected applications as needed.

Consultants are required to absent themselves from the room during the review of any application if their presence would constitute or appear to constitute a conflict of interest.
